# Prolonged anoxic exposure impacts antibiotic sensitivity profiles of *Pseudomonas aeruginosa*

**DOI:** 10.1093/femsle/fnaf066

**Published:** 2025-07-03

**Authors:** Maik Kok, Wisse van Os, Thomas Hankemeier, J G Coen van Hasselt

**Affiliations:** Leiden Academic Centre for Drug Research, Leiden University, Einsteinweg 55, 2333 CC Leiden, The Netherlands; Leiden Academic Centre for Drug Research, Leiden University, Einsteinweg 55, 2333 CC Leiden, The Netherlands; Leiden Academic Centre for Drug Research, Leiden University, Einsteinweg 55, 2333 CC Leiden, The Netherlands; Leiden Academic Centre for Drug Research, Leiden University, Einsteinweg 55, 2333 CC Leiden, The Netherlands

**Keywords:** anoxia, antibiotic sensitivity, metabolic specialization, oxygen, *Pseudomonas aeruginosa*

## Abstract

Chronic respiratory tract infections with *Pseudomonas aeruginosa* frequently occur in patients with cystic fibrosis, chronic obstructive pulmonary disease, and bronchiectasis. A hallmark of these conditions is the accumulation of mucus plugs, creating oxygen-limited niches. Within these microenvironments, *P. aeruginosa* undergoes cellular modifications that may alter its antibiotic sensitivity. Although the acute effects of anoxia are well studied, the impact of prolonged anoxic exposure on antibiotic sensitivity remains unclear. In this study, we developed anoxic-conditioned *P. aeruginosa* strains by passaging a laboratory strain for 22 days in an anoxic environment. We performed time-kill assays with both parental and anoxic-conditioned strains in anoxic and aerobic environments, using ceftazidime, ciprofloxacin, colistin, and tobramycin. The anoxic-conditioned strains exhibited increased susceptibility to tobramycin and reduced sensitivity to colistin and ceftazidime. These differences were attributed to altered killing rates (as with tobramycin) or reduced regrowth under anoxic conditions (as with colistin). For ciprofloxacin, a steeper killing rate was observed against the anoxic-conditioned strains, but 24-h outcomes were similar to the parental strain. Overall, our findings demonstrate that long-term anoxia alters antibiotic sensitivity in *P. aeruginosa* differently than acute anoxia, with important implications for treating chronic infections in oxygen-limited environments.

## Teaser

A study of the effects of prolonged *Pseudomonas aeruginosa* adaptation to oxygen-limited environments on antibiotic sensitivity.

## Introduction

Lung diseases such as chronic lung conditions such as cystic fibrosis (CF), chronic obstructive pulmonary disease, and non-CF bronchiectasis (Worlitzsch et al. 2002, Boucher 2019) are characterized by anoxic environments. These anoxic environments are caused by airway narrowing and the buildup of thick, dehydrated mucus limit oxygen penetration, and high oxygen consumption of the host immune response further exacerbates anoxia (Sønderholm et al. 2017). In these anoxic niches, the respiratory pathogen *Pseudomonas aeruginosa* uses fermentation and alternative electron acceptors to sustain cellular functions and proliferation (Eschbach et al. 2004, Palmer et al. 2007). These metabolic adaptations that support survival of *P. aeruginosa* in anoxic niches can also influence its sensitivity to antibiotics. Antibiotics commonly used against *P. aeruginosa*, including tobramycin, ciprofloxacin, and ceftazidime, rely on oxygen-dependent processes—such as oxidative phosphorylation, reactive oxygen species production, and active bacterial proliferation—to exert their antimicrobial effects (Crabbé et al. 2019). In contrast, colistin remains effective in anoxic environments because energy-starved cells are unable to modify their membranes to reduce colistin binding (Pamp et al. 2008).

While the differential impact of acute responses to anoxic conditions on antibiotic sensitivity have been previously studied, the consequences of prolonged anoxia-induced adaptations remain largely unknown. Longer-term genetic or transient changes in cellular metabolism, stress responses, biofilm formation, and membrane remodeling have yet to be characterized in detail (Wu et al. 2005, Fang et al. 2013, Kamath et al. 2017). Alterations in membrane composition and transport proteins may affect the uptake and effect of membrane-targeting antibiotics such as ceftazidime and colistin. Additionally, metabolic specialization to anoxia introduces cellular redox imbalances that can alter antibiotic activity (Glasser et al. 2014). In parallel, the development of oxygen intolerance due to anoxic specialization can lead to higher levels of reactive oxygen species (ROS) upon re-exposure to oxygen (Kvich et al. 2019). These prolonged metabolic adaptations can influence the sensitivity of ROS-dependent antibiotics such as ciprofloxacin and metabolism-dependent antibiotics such as tobramycin. Thus, long-term anoxic adaptation affects key mechanisms relevant to antibiotic activity, but the potential impact on antibiotic sensitivity remains unclear.

In this study, we investigated how prolonged anoxic exposure affects the sensitivity of *P. aeruginosa* to ceftazidime, ciprofloxacin, colistin, and tobramycin. An anoxic-conditioned strain was obtained by passaging a *P. aeruginosa* laboratory strain in an anoxic environment (<1% oxygen) for 22 days. The effects of the antibiotic-classes were then compared between the conditioned and parental strains under both anoxic and atmospheric oxygen conditions using time-kill assays, providing a comprehensive view of how anoxic specialization influences antibiotic sensitivity.

## Materials and methods

### Strains, culture media, agar plates, and antibiotics

*Pseudomonas aeruginosa* PAO1 (DSM1117) was used as the parental strain in this study. Synthetic cystic fibrosis sputum medium (SCFM) served as the liquid medium for anoxic conditioning and time-kill experiments (Table S1) (Palmer et al. 2007). Samples for colony-forming unit (CFU) enumeration were diluted using a 1:4 dilution of SCFM with 0.11 M phosphate buffer before plating. Agar plates were prepared with Mueller–Hinton broth agar supplemented with 10 mM KNO₃ (Acros Organics, Geel, Belgium) to prevent the loss of oxygen-intolerant populations during aerobic plating.

Antibiotic solutions were prepared and diluted with SCFM to the desired concentrations in microtiter plates using an Opentrons OT-2 liquid handling system (Opentrons Inc., New York, NY, USA) 1 day before the time-kill assay. Ceftazidime pentahydrate was purchased from Thermo Fisher Scientific (Breda, The Netherlands), ciprofloxacin and tobramycin from Chem-Impex International (Wood Dale, IL, USA), and colistin sulfate from Cayman Chemical Company (Ann Arbor, MI, USA).

### Anoxic culture environment and anoxic conditioning

Anoxic cultures were performed in a Baker Ruskin anoxic workstation (Sanford, ME, USA). All liquid media used for anoxic experiments were pre-conditioned to the anoxic environment (<1% oxygen) for 2 days, and antibiotic microtiter plates were placed in the anoxic chamber 1 day before the time-kill assay.

Anoxic-conditioned strains were generated in triplicate, each from randomly selecting colonies of the parental strain grown overnight on an aerobic agar plate culture. Conditioning was conducted in triplicate using 2 ml pre-acclimated SCFM medium in sealed 12 ml culture tubes at 37°C in the anoxic chamber. Every two days, 20 µl of the cultures was transferred into fresh SCFM, continuing for 22 days to achieve anoxic conditioning.

### Time-kill assay

Time-kill assays in SCFM were performed in triplicate for the parental and anoxic-conditioned *P. aeruginosa* strains in aerobic and anoxic environments to examine antibiotic sensitivity (Fig. S1). The parental strain replicates were subcultured for 20 h under aerobic conditions, while the anoxic conditioned strains were used directly following the 22-day incubation period. Liquid cultures from the parental and anoxic-conditioned strain were diluted to reach a starting density of ∼1 × 10^7^ CFU/ml, based on optical density measurements at 600 nm. Cultures were exposed to five antibiotic concentrations in two-fold serial dilutions, along with an antibiotic-free control. Antibiotic concentrations for the assays were guided by visual minimum inhibitory concentration (MIC) testing of the parental strain performed aerobically (Table S2). MIC determination under anoxic conditions was not feasible due to low final culture densities.

Anoxic time-kill assays were incubated within the anoxic chamber, while aerobic assays were incubated at 37°C with shaking (250 rpm) to ensure oxygenation. At 2 and 24 h, 100 µl samples were taken, subjected to 10-fold serial dilutions, and plated on agar. All agar plates were incubated under aerobic conditions at 37°C for 1 day, followed by an additional day at room temperature, before colony counting to determine bacterial population size.

### Growth rate assay

Growth curves were obtained in quadruplicate in both aerobic and anoxic culture environments for the anoxic-conditioned and parental *P. aeruginosa* strains to compare antibiotic-free growth kinetics. Growth curves in the aerobic environment were obtained by transferring microtiter plates every 30 min between a Liconic StoreX STX44 incubator (Mauren, Liechtenstein) shaking at 150 rpm and a BMG Labtech Fluostar Omega microplate reader (Ortenberg, Germany), using a Peak Analysis and Automation KX-2 laboratory robot (Hampshire, UK). Growth in the anoxic environment was measured every 30 min using a wireless Cerillo Alto plate reader (Charlottesville, VA, USA).

### Data processing

Drug effects were quantified by calculating the change in bacterial population size over 24 h of antibiotic exposure. To evaluate the initial bacterial response, the antibiotic net killing rate during the first 2 h was determined by calculating the slope using the logarithmically transformed population sizes (N) and the elapsed time (Δ*t*) (Equation 1). Maximal growth rates in antibiotic-free media were calculated using the splines function from the *growthrates* package in R, based on the growth curves from four technical replicates. All data analysis was performed in R (version 4.3.0)


(1) \begin{eqnarray*}
\textit{Killing}\ \textit{rate} = \ \frac{{lo{{g}_{10}}\left( {{{N}_2}} \right) - {\mathrm{\ }}lo{{g}_{10}}\left( {{{N}_1}} \right)}}{{\Delta t}}.
\end{eqnarray*}

## Results

### Acute and prolonged anoxic exposures modulate antibiotic effect over 24 h

**Figure 1.**
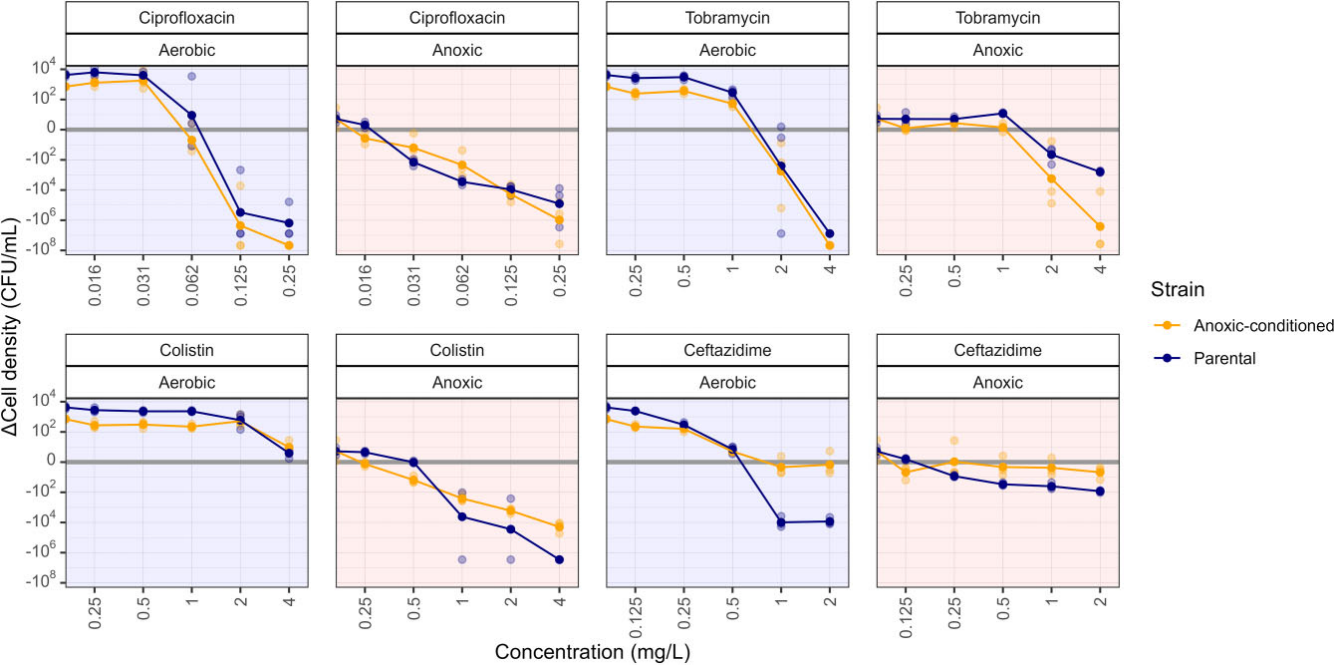
Change in cell density of the parental and anoxic-conditioned *P. aeruginosa* strains after 24 h of antibiotic exposure in aerobic and anoxic environments. Orange lines and points represent the anoxic-conditioned *P. aeruginosa* strain, evolved over 22 days in an anoxic environment prior to antibiotic exposure, while navy lines and points represent the parental PAO1 strain. Solid lines and points denote the mean log_10_ change in cell density, calculated from the three biological replicates, which are represented as translucent points.

We investigated how anoxic conditions affect antibiotic activity by comparing time-kill curves obtained in aerobic and anoxic environments, both for the parental and the anoxic-conditioned strains. Time-kill experiments with the parental strain were performed to evaluate the acute effects of anoxic environments, whereas experiments with the anoxic-conditioned strain reflected prolonged adaptation to anoxia. We compared the change in bacterial population size between the inoculum and the 24-h timepoint as a measure of antibiotic effect (Fig. 1).

For ciprofloxacin, population size reduction of the parental strain was observed at 0.031 mg l^−1^ in anoxic environments, while 0.125 mg l^−1^ was required in aerobic environments. The anoxic-conditioned strain showed a similar oxygen-dependent response to ciprofloxacin but at one serial-dilution step lower (0.016 mg l^−1^ in anoxic and 0.062 mg l^−1^ in aerobic environments).

With tobramycin, population sizes of the parental strain started to decline after 24 h at concentrations above 1 mg l^−1^, with a notably more pronounced reduction at 4 mg l^−1^ under aerobic compared to anoxic conditions. At these concentrations, the anoxic-conditioned strain mirrored the aerobic response of the parental strain in both oxygen environments.

For both strains, all evaluated colistin concentrations failed to induce net reductions in population size in aerobic environments. However, reductions were observed in anoxic environments, with a smaller reduction for the anoxic-conditioned strain.

For ceftazidime, a flat concentration–effect relationship was observed in anoxic environments for both strains. In the aerobic environment, on the other hand, a clear concentration–effect relationship was observed for the parental strain, with population size reductions above 1 mg l^−1^. In contrast, the highest tested ceftazidime concentration failed to reduce the population size of the conditioned strain in the aerobic environment.

### Anoxic conditioning impacts antibiotic net killing rates

**Figure 2.**
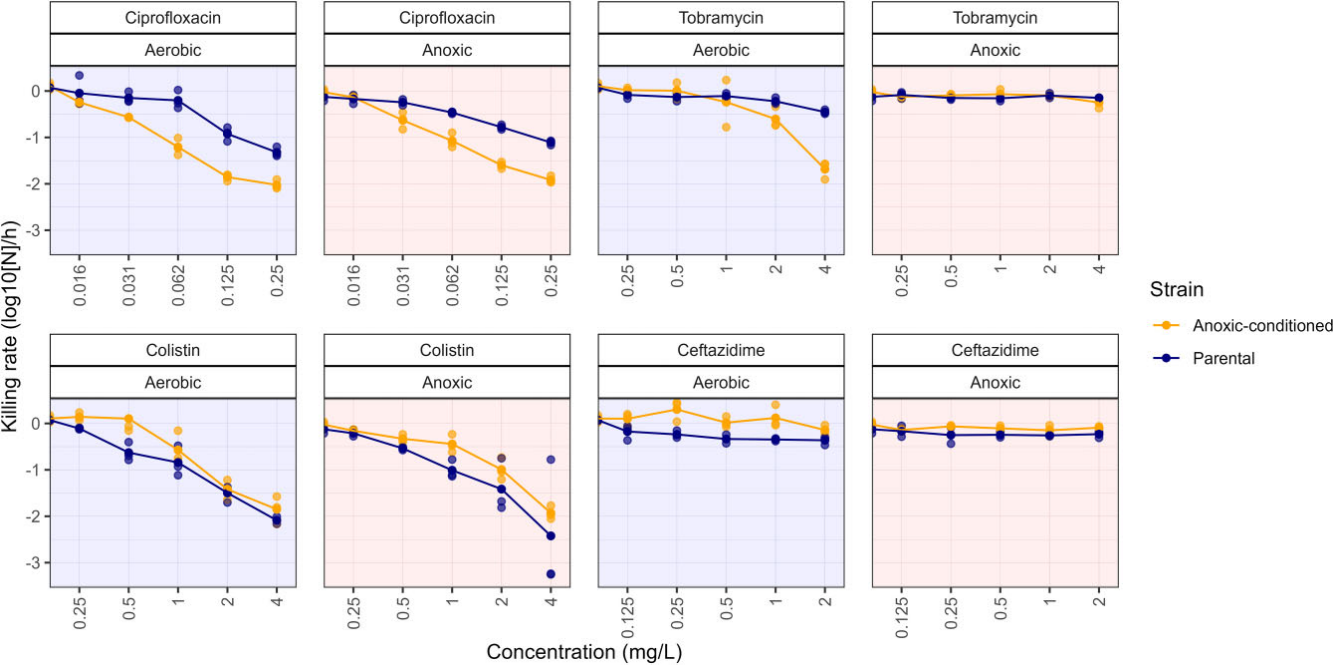
Net antibiotic killing rates in parental and anoxic-conditioned *P. aeruginosa* under aerobic and anoxic environments. Net killing rates during the initial 2 h of antibiotic exposure for each biological replicate (shown as points) were calculated by determining the slope of the change in log_10_-transformed cell density. Solid lines represent the mean response rate of the mean parental PAO1 strain as well as the conditioned *P. aeruginosa* strain, which was evolved in an anoxic environment for 22 days prior to treatment.

To assess the effect of anoxic conditioning on the immediate bacterial response to antibiotics, we analyzed net killing rates during the first 2 h of antibiotic exposure (Fig. 2). The net killing rate was defined as the slope of the log_10_-transformed bacterial population size over time (log_10_[Δ*N*]/*t*), with steeper negative slopes indicating faster killing. For ciprofloxacin, faster killing rates were observed at concentrations of 0.031 mg l^−1^ and above for the anoxic-conditioned strain compared to the parental strain, in both aerobic and anoxic culture environments. Tobramycin demonstrated consistent effects across most conditions, except for the anoxic-conditioned strain in aerobic environments, for which the fastest killing rate of −1.68 (corresponding to a 50-fold reduction per hour) was observed at 4 mg l^−1^, compared to −0.45 for the parental strain (2.8-fold reduction). For colistin, similar killing rates between strains and oxygen environments were observed, with rates becoming more negative at higher concentrations. The largest magnitude of killing (−2.42, corresponding to a 263-fold reduction per hour) was observed at 4 mg l^−1^ in the parental strain under anoxic conditions. By contrast, ceftazidime had minimal killing across all strains and environments, with a fastest killing rate of −0.36 (a 2.3-fold reduction per hour).

### Prolonged anoxic exposure reduces bacterial growth rates

**Figure 3.**
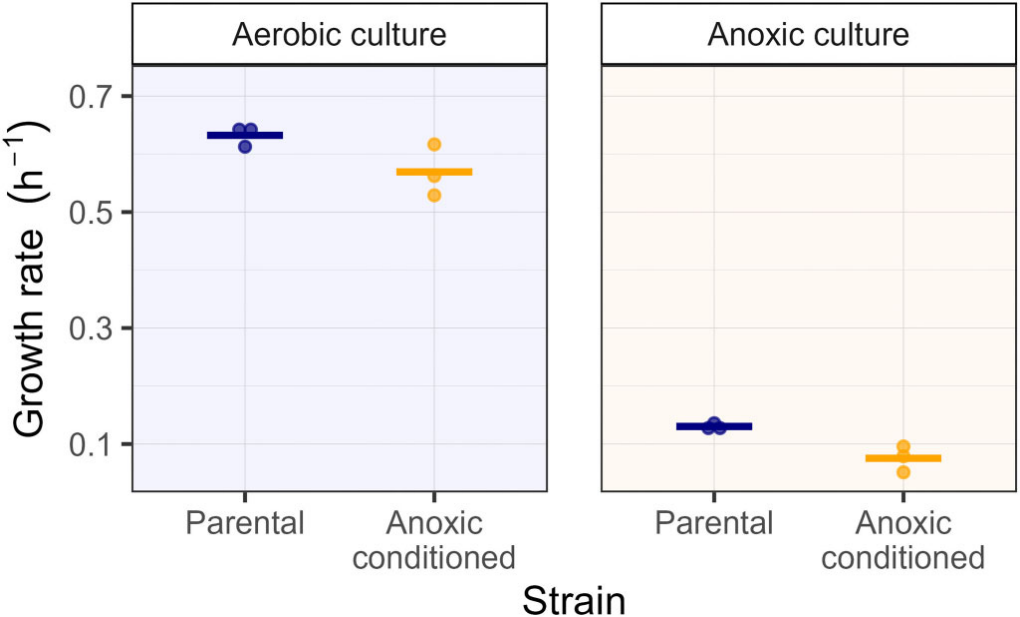
Growth rates of the parental and anoxic-conditioned *P. aeruginosa* strains in aerobic and anoxic environments. Maximal growth rates were calculated from smoothed spline growth curves of four technical replicates per strain and condition. The growth curve is based on optical density at 600 nm (OD600) measurements taken every 30 min. Data points represent µmax values per biological replicate, and the solid line indicates the mean µmax of the three biological replicates.

To assess fitness differences between the parental strain and anoxic-conditioned strains, we derived the maximal growth rates under antibiotic-free conditions. Here, the growth rate reflects the time a strain requires for doubling its population size. The growth rate of the parental strain was on average 4.9-fold increase in aerobic than in anoxic environments, and for the anoxic-conditioned strain it was 8.1-fold increased (Fig. 3). A moderate increase in growth rate was observed for the parental strain than for the anoxic-conditioned strain in both aerobic (0.63 h^−1^ vs. 0.57 h^−1^) and anoxic (0.13 h^−1^ vs. 0.07 h^−1^) environments.

## Discussion

In this study, we demonstrated how prolonged adaptation of a *P. aeruginosa* laboratory strain to anoxic environments reduces bacterial growth rates and alters antibiotic susceptibility in a class-dependent manner. Anoxic specialization resulted in increased effect of tobramycin, reduced effects of colistin and ceftazidime, and a higher initial killing rate for ciprofloxacin. In the aerobic environment, ciprofloxacin and tobramycin exhibited increased initial killing rates against the anoxic-conditioned strain whereas ceftazidime effects after 24 h of exposure were decreased.

The limited difference in ciprofloxacin effects over 24 h exposure between anoxic and aerobic environments aligns with previous findings that oxygen-deprived environments primarily induce fluoroquinolone tolerance rather than altering antibiotic susceptibility (King et al. 2010, Gupta et al. 2016). The steeper killing rate observed in the anoxic-conditioned strain under aerobic conditions is consistent with the dependence of fluoroquinolones on reactive oxygen species for activity (Gutierrez et al. 2018). Oxygen radicals are formed in greater quantities when oxygen is introduced to cells that have undergone anoxic specialization (Kvich et al. 2019). The increased ciprofloxacin effect observed in the anoxic-conditioned strain in the anoxic environment suggests that additional biological adaptations to anoxia may influence fluoroquinolone effects.

Anoxic conditioning increased tobramycin sensitivity in both aerobic and anoxic environments, contrasting with the established reliance of aminoglycosides on the proton motive force for intracellular entry (Taber et al. 1987). Although oxygen-deprived environments typically exhibit lower membrane potentials, active transport systems can still facilitate aminoglycoside uptake (Lang et al. 2023). Once inside, aminoglycosides trigger secondary stress responses, such as envelope and oxidative stress, potentially explaining the enhanced 24-h tobramycin effect on the anoxic-conditioned strain. Despite similar 24-h outcomes between the strains in the aerobic environment, a substantially higher killing rate was observed in the anoxic-conditioned strain compared to the parental strain, highlighting the impact of prolonged anoxic adaptation on aminoglycoside activity. These observations align with reports of pronounced differences in aminoglycoside sensitivity in clinical CF respiratory tract isolates across oxygen gradients, likely due to prior exposure to anoxic environments (Field et al. 2005, King et al. 2010, Gupta et al. 2016).

The drug effect of colistin after 24 h of exposure was stronger in the anoxic environment for both strains, consistent with previous findings that oxygen deprivation enhances colistin activity (Pompilio et al. 2015, Kolpen et al. 2016). The increased net effect in anoxic conditions may be due to lower bacterial growth rates in this environment, which can result in higher net effect or reduced regrowth. Notably, the killing rates did not substantially differ between oxygen conditions, and the anoxically conditioned strain showed a reduced colistin effect. This suggest a more complex interplay between colistin’s mechanism of action and *P. aeruginosa* responses under varying oxygen levels. *Pseudomonas aeruginosa* is known to modify the lipopolysaccharide composition of its membrane (Lam et al. 2011), the primary target of colistin (Lo Sciuto et al. 2020), as a resistance mechanism when energy supplies are sufficient (Pamp et al. 2008). This may explain the regrowth observed in an aerobic culture environment. Currently, nebulized colistin is extensively evaluated as an adjunctive therapy to prevent ventilator-associated pneumonia (VAP) in mechanically ventilated patients (Zhang et al. 2023). Our results suggest that increased lung oxygenation through ventilation might negatively affect colistin efficacy, potentially contributing to the limited effectiveness of prophylactic colistin treatment in reducing VAP incidence (Karvouniaris et al. 2015). Colistin exhibits greater clinical efficacy in CF respiratory infections, where anoxic microenvironments are common (Stilma et al. 2018). However, the role of anoxia in lipopolysaccharide modification remains poorly understood, such modifications may also explain the reduced colistin effects against the anoxic-conditioned strain. Investigating these modifications further represents an important direction for future research.

Ceftazidime displayed a distinct pharmacodynamic profile compared to the other antibiotics tested. No differences in killing rates were observed during the initial hours of exposure, which is consistent with the time-dependent activity of ceftazidime (Muller and Mouton 2014). However, over the full 24-h period, ceftazidime sensitivity was markedly reduced following anoxic conditioning, both under aerobic and anoxic assay environments. Although ceftazidime is typically bactericidal for *P. aeruginosa* (Noel et al. 2022), this effect was only evident in the parental strain, albeit to a lesser degree in anoxic conditions. For the anoxic-conditioned strains, the effect of ceftazidime was limited to bacteriostasis in both aerobic and anoxic environments. This reduction in activity may be explained by previous work showing that oxygen deprivation enhances efflux pump activity (Schaible et al. 2012). Reduced ceftazidime sensitivity in anaerobic biofilms has been reported (Bowler et al. 2012), and our results demonstrate that this effect persists even after re-culturing under aerobic conditions.

We adopted a 22-day adaptation period to induce anoxic specialization. We compared the anoxically conditioned strain directly to the unpassaged parental strain, rather than to a 22-day aerobically subcultured strain. This choice was deliberate, as prolonged aerobic propagation would introduce substantially different genetic drift due to markedly higher growth rates and cell densities compared to the anoxic culture environment. While we acknowledge the limitations of this control setup, it allowed us to more accurately attribute observed differences to direct anoxic adaptation resulting from prolonged conditioning. Additionally, we recognize that the 22-day anoxic incubation used here is considerably shorter than typically experienced by *P. aeruginosa* strains persistently present in the CF lung, where prolonged evolutionary pressures may drive further specialization (La Rosa et al. 2019). The current study utilized *P. aeruginosa* PAO1 laboratory strain to specifically study the effects of anoxic specialization on antibiotic response. Clinical isolates exhibit diverse adaptations due to prolonged in-host evolution, potentially affecting responses to anoxic environments. Using a defined laboratory strain ensures consistent genetic and phenotypic backgrounds, facilitating clearer interpretations of the observed antibiotic responses. Although beyond the scope of this study, future research involving clinical isolates represents a logical and necessary next step. We anticipate that such studies will require larger sample sizes to address the inherent heterogeneity among isolates, and they will greatly benefit from the experimental framework and treatment conditions established here. Finally, investigations into membrane modifications and redox imbalances could yield deeper mechanistic insights to inform the optimization of treatment strategies.

In conclusion, our findings show that anoxic adaptation of a *P. aeruginosa* laboratory strain modifies the effects of ceftazidime, ciprofloxacin, colistin, and tobramycin differently compared to acute anoxic exposure of the parental strain. Ciprofloxacin and tobramycin became more effective, whereas colistin and ceftazidime exhibited reduced effects against the anoxic-conditioned strain. These altered antibiotic effects were also observed under aerobic conditions, suggesting sustained anoxia-induced cellular adaptations that alter antibiotic sensitivity. These findings highlight the importance of considering oxygen gradients in research aimed at optimizing antibiotic treatment for chronic *P. aeruginosa* infections in the CF lung.

## Supplementary Material

fnaf066_Supplemental_File
