# Supplementary material for: Prolonged anoxic exposure impacts antibiotic sensitivity profiles of Pseudomonas aeruginosa
Source: FEMS Microbiol Lett. 2025 Jul 3;372:fnaf066. doi: 10.1093/femsle/fnaf066 (PMC12254951; doi:10.1093/femsle/fnaf066)
Supplement: fnaf066_Supplemental_File [file fnaf066_supplemental_file.docx]

**Supplementary Materials**

**Supplemental table 1.** Detailed content list of synthetic cystic fibrosis sputum media

|  | **Name** | **Concentration (mM)** | | **Company information** |
| --- | --- | --- | --- | --- |
| M9 buffer | di-sodium hydrogen phosphate (Na_2_HPO_4_) | 90.2 | Thermo Fisher Scientific , Breda, The Netherlands | |
|  | Potassium di-hydrogen phosphate (KH_2_PO_4_) | 22.0 | VWR International, Amsterdam, The Netherlands | |
|  | Sodium chloride (NaCl) | 8.5 | Merck KGaA (Avantor™), Darmstadt, Germany | |
|  | Ammonium chloride (NH_4_Cl) | 18.6 | Alfa Aesar (Thermo Fisher GmbH), Kandel, Germany | |
|  | Magnesium sulphate hepta-hydrate (MgSO_4_) | 1.0 | VWR International, Amsterdam, The Netherlands | |
|  | Calcium chloride (CaCl_2_) | 0.1 | Acros Organics (Thermo Fisher Scientific), Geel, Belgium | |
| Salts | Potassium nitrate (KNO_3_) | 0.35 | Acros Organics (Thermo Fisher Scientific), Geel, Belgium | |
|  | Iron sulphate (FeSO_4_) | 0.0036 | Alfa Aesar (Thermo Fisher GmbH), Kandel, Germany | |
| Vitamins | BME Vitamin solution | *1x* | Thermo Fisher Scientific , Breda, The Netherlands | |
| Trace metals | Di-sodium Ethylene di-amine tetra-acetic acid (EDTA) | 0.002 (mg/mL) | J.T. Baker (Avantor™), Darmstadt, Germany | |
|  | Zinc Sulphate hepta-hydrate (ZnSO_4_) | 0.23 (mg/mL) | Alfa Aesar (Thermo Fisher GmbH), Kandel, Germany | |
|  | Boric acid (H_3_BO_3_) | 0.111 (mg/mL) | Acros Organics (Thermo Fisher Scientific), Geel, Belgium | |
|  | Manganese chloride tetra-hydrate (MnCl_2_) | 0.051 (mg/mL) | Sigma Aldrich (Avantor™), Darmstadt, Germany | |
|  | Cobalt chloride (CoCl_2_) | 0.017 (mg/mL) | Alfa Aesar (Thermo Fisher GmbH), Kandel, Germany | |
|  | Copper Sulphate penta-hydrate (CuSO_4_) | 0.015 (mg/mL) | Sigma Aldrich (Avantor™), Darmstadt, Germany | |
|  | Ammonium hepta-molybdate tetra hydrate ((NH_4_)_6_ Mo_7_O_2_) | 0.01 (mg/mL) | Alfa Aesar (Thermo Fisher GmbH), Kandel, Germany | |
| Basis nutrients | Alanine (Ala) | 1.8 | Chem-Impex International, Wood Dale, IL, USA | |
|  | Arginine (Arg) | 0.3 | Chem-Impex International, Wood Dale, IL, USA | |
|  | Aspartate (Asp) | 0.8 | Chem-Impex International, Wood Dale, IL, USA | |
|  | Cysteine (Cys) | 0.2 | Chem-Impex International, Wood Dale, IL, USA | |
|  | Glucose (GLC) | 3.2 | Alfa Aesar (Thermo Fisher GmbH), Kandel, Germany | |
|  | Glutamate (Glu) | 1.5 | Chem-Impex International, Wood Dale, IL, USA | |
|  | Glycine (Gly) | 1.2 | Acros Organics (Thermo Fisher Scientific), Geel, Belgium | |
|  | Histidine hydrochloride (His) | 0.5 | Chem-Impex International, Wood Dale, IL, USA | |
|  | Isoleucine (Ile) | 1.1 | Chem-Impex International, Wood Dale, IL, USA | |
|  | Lactate (LAC) | 9.0 | Biosynth International, Compton, United Kingdom | |
|  | Leucine (Leu) | 1.6 | Chem-Impex International, Wood Dale, IL, USA | |
|  | Lysine hydrochloride (Lys) | 2.1 | Thermo Fisher Scientific , Breda, The Netherlands | |
|  | Methionine (Met) | 0.6 | Chem-Impex International, Wood Dale, IL, USA | |
|  | Phenylalanine (Phe) | 0.5 | Chem-Impex International, Wood Dale, IL, USA | |
|  | Proline (Pro) | 1.7 | Thermo Fisher Scientific , Breda, The Netherlands | |
|  | Serine (Ser) | 1.4 | Chem-Impex International, Wood Dale, IL, USA | |
|  | Threonine (Thr) | 1.0 | Chem-Impex International, Wood Dale, IL, USA | |
|  | Tryptophan (Trp) | 0.01 | Chem-Impex International, Wood Dale, IL, USA | |
|  | Tyrosine (Tyr) | 0.8 | Chem-Impex International, Wood Dale, IL, USA | |
|  | Valine (Val) | 1.1 | Chem-Impex International, Wood Dale, IL, USA | |

**Supplemental table 2.** Median minimal inhibitory concentrations (MIC) of antibiotics for the parental *P. aeruginosa* strain (n=3 biological replicates). MICs were determined after 20 hours of aerobic incubation at 37 °C with shaking (250 rpm).

| **Strain** | **Ceftazidime** | **Colistin** | **Ciprofloxacin** | **Tobramycin** |
| --- | --- | --- | --- | --- |
| **PAO1 (DSM1117)** | 0.5 mg L⁻¹ | 4 mg L⁻¹ | 0.125 mg L⁻¹ | 1 mg L⁻¹ |

**
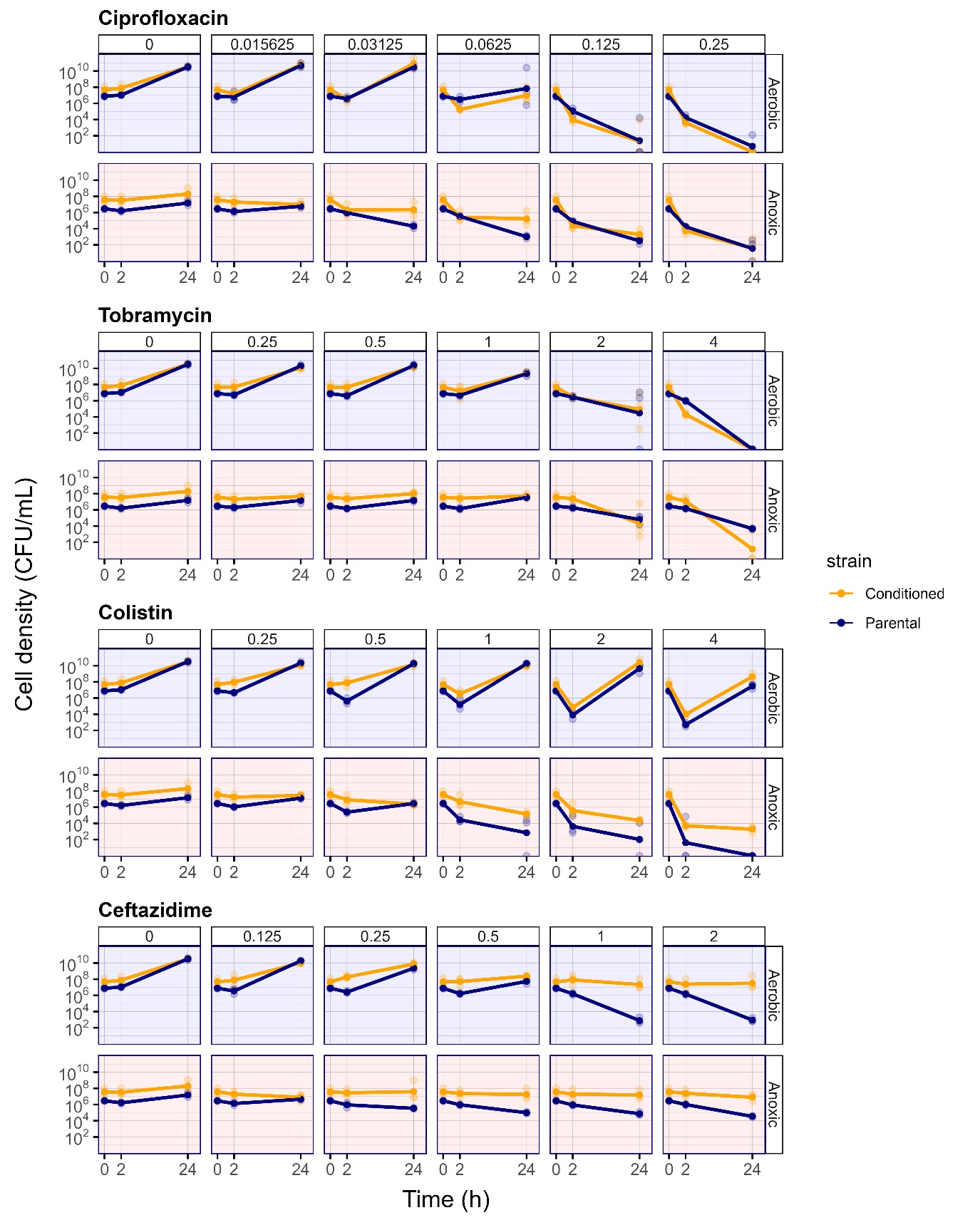
**

**Supplemental figure 1. Antibiotic time-kill assays comparing parental and anoxic conditioned *P. aeruginosa* under anoxic and aerobic conditions**. Mean cell densities over time are shown with solid navy blue points and lines for the parental PAO1 strain, and orange points and lines for the anoxic conditioned strain. The means were calculated from the log-transformed cell densities of three biological replicates, represented by translucent points. Background shading indicates treatment conditions, with blue for aerobic and orange for anoxic environments.
